# Young People's Views on Accelerometer Use in Physical Activity Research: Findings from a User Involvement Investigation

**DOI:** 10.5402/2012/948504

**Published:** 2012-11-07

**Authors:** Joanna Kirby, Carly Tibbins, Claire Callens, Beckie Lang, Margaret Thorogood, William Tigbe, Wendy Robertson

**Affiliations:** ^1^Division of Health Sciences, Warwick Medical School, University of Warwick, Coventry CV4 7AL, UK; ^2^MCRN User Involvement, Birmingham Children's Hospital, Birmingham B4 6NH, UK

## Abstract

The use of accelerometers to objectively measure physical activity is important in understanding young people's behaviours, as physical activity plays a key part in obesity prevention and treatment. A user-involvement qualitative study with young people aged 7–18 years (*n* = 35) was carried out to investigate views on accelerometer use to inform an obesity treatment research study. First impressions were often negative, with issues related to size and comfort reported. Unwanted attention from wearing an accelerometer and bullying risk were also noted. Other disadvantages included feeling embarrassed and not being able to wear the device for certain activities. Positive aspects included feeling “special” and having increased attention from friends. Views on the best time to wear accelerometers were mixed. Advice was offered on how to make accelerometers more appealing, including presenting them in a positive way, using a clip rather than elastic belt to attach, personalising the device, and having feedback on activity levels. Judgements over the way in which accelerometers are used should be made at the study development stage and based on the individual population. In particular, introducing accelerometers in a clear and positive way is important. Including a trial wearing period, considering practical issues, and providing incentives may help increase compliance.

## 1. Introduction

The benefits of physical activity among young people are well documented. Evidence shows physical inactivity during childhood to be associated with higher risk of childhood obesity, as well as being a risk factor for cardiovascular disease, cancer, and osteoporosis in later life [1]. Physical activity has an important role in body weight regulation, both in preventing weight gain and maintaining weight loss over time [2]. As such, understanding young people's physical activity behaviours is important in promoting participation among this group, particularly when activity patterns may vary between lean and obese children [3].

In order to establish physical activity participation levels, accurate measurement is required. Objective measures are being increasingly used to quantify the amount and intensity of physical activity, as well as levels of sedentary behaviour, with accelerometers currently being the favoured objective measuring device [4]. Accelerometers have been used in studies to measure general physical activity levels among young people [5, 6], as well as in conjunction with measurement of body composition [7] or alongside a health intervention [8]. Although publications relating to accelerometer data analyses are common [9, 10], those reporting findings related to practical issues in data collection are less so [11, 12].

The acceptability to young people of wearing accelerometers has previously been assessed as good, based on low study dropout rates from children not wanting to wear the monitor [13]. However, previous studies found some parents reporting that their children were unwilling to wear accelerometers at school and during sports due to the risk of stigma and bullying [8]. There is limited qualitative research on how young people themselves feel about wearing accelerometers for physical activity research. Investigations of adults' views on wearing accelerometers have shown noncompliance to be related to occupational factors or discomfort during sitting or driving [14]. However, these are adult-specific issues, and different reasons for noncompliance are likely to be relevant to children. One recent study [12] investigated the use of accelerometers among adolescents in relation to recruitment, retention, and adherence to the study protocol. The authors reported that adherence may be improved by following a two-part reward system, whereby part one is for returns and part two is for adherence. The use of personal activity graphs and less obtrusive belts and monitors was also suggested. A review on the use of accelerometers in field-based research suggests that researchers need to devise a plan to promote compliance with the monitoring protocol and minimise the possibility of acquiring incomplete data [15]. The authors suggest that further information is required to identify the psychological, sociocultural, and environmental factors that are associated with or predict compliance among individuals wearing accelerometers. The importance of young people's participation at the onset of the research process is well recognised and advocated by professional bodies [16, 17]. User involvement in research has been interpreted as a shift away from assumptions that “experts” are the best judges [18]. High quality research and good retention rates depend on listening to the voices of children and young people themselves and taking account of their experiences, priorities, and perspectives [19].

This paper presents findings from a user involvement study with young people, aged 7–18 years. The data form part of a larger user involvement study, aimed at informing a proposed obesity interventional randomised control trial. This paper reports data sought about accelerometer use. It presents the practicalities of using accelerometers within the research setting from young people's perspective and, based on these findings, goes on to offer practical advice to researchers using accelerometers with this group.

## 2. Methods

A total of five focus group discussions took place; involving 35 young people aged 7–18 years and facilitated by two user involvement coordinators from the West Midlands Medicines for Children Research Network (MCRN) (CC, CT). Participants comprised members of two MCRN young person's advisory groups (YPAG) (groups 1-2) and pupils (groups 3–5) from two local primary schools. Group 2 was made up predominantly of young people who were overweight. MCRN young person's advisory groups have previously been shown to be effective in providing a forum for young people to learn about and comment on various aspects of the research cycle [19]. Focus groups with young people allow for differences between participants to be revealed and are considered particularly useful in attitudinal research and when interaction between participants can help to illuminate a research issue [20]. The MCRN young person's advisory group had previously taken part in a weight-related trial which meant that they formed a particularly appropriate group to give advice on the use of accelerometers. The two schools were selected from locations where the obesity interventions were to take place, and in liaison with school staff, pupils were selected to take part in a focus group discussion. Participants included both boys and girls and a range of ethnicities and weight status. A broad range of ages were included, to identify differences in views across the age span. Written consent was obtained from parents in advance of the research. Verbal consent was attained from all participating young people. Prior to participation in focus group discussions, some participants (*n* = 18) were given the opportunity to wear an accelerometer (GT1M Actigraph, Fort Walton, FL) for a given time in order to inform the discussion. The accelerometer was worn around the waist using an elastic belt. Details of the groups and main focus group discussion topic are shown in Table 1.

The researchers took notes throughout the FGDs or wrote participants' thoughts on a flipchart. These were later analysed using thematic analysis [21]; based on what themes emerged and how often these themes were discussed. Ethical approval was obtained from the NRES Committee West Midlands-Coventry and Warwickshire, REC (Reference 11/WM/0290).

## 3. Results

Five broad themes were drawn out from the discussions. Although it is acknowledged that there is overlap across the themes, for the purposes of data presentation, these are reported as follows.

### 3.1. First Impressions

The majority of first impressions on accelerometers were negative. For younger schoolchildren (7–11 years) the main concern was a feeling that the accelerometer was “watching” them, believing it to have a camera or microphone contained within it. This was present even after the researcher had assured them this was not the case. Across the full age range (7–18 years) many expressed a general dislike in the physical appearance of accelerometers, mainly around the size and appearance, describing them as being too big and bulky. Words used to describe the devices included “unattractive,” “too thick,” and “not nice.” The elastic waistband onto which the accelerometer was attached was also described as being uncomfortable.

### 3.2. How It Feels to Wear an Accelerometer

Some positive aspects of wearing an accelerometer were noted, for example, feeling “different” or “special” in some way. Several young people stated that they were constantly asked questions about the accelerometers by their peers, who also wanted to be involved or try them on.

*“I wanted my friends to be jealous that I had something different.”* (girl, aged 10; wore accelerometer 1 week)**

*“After a while I got used to it [accelerometer], so it did not annoy me. Only one child asked me about it but was very interested and understanding about why I needed to wear it.”* (girl, aged 10; wore accelerometer 1 week)**

Some reported that it took them a little while to get used to wearing the device, either taking it on and off several times when initially worn, or simply taking some time to forget they had it on.

*“It took me a while to get used to having it on, so the first day I took it on and off several times.”* (girl aged 10; wore accelerometer 1 week)**

Among children (7–11 years) who wore the accelerometer for one school day (*n* = 12), half felt constantly reminded they had them on, while the other half forgot. Girls appeared to be more self-conscious and aware of wearing them. Some reported finding them distracting to wear and constantly wanted to touch the accelerometer. In general, all age groups expressed a preference to wearing accelerometers under rather than over their clothes, although three pupils (all girls) did not mind and one (boy) felt strongly that he should wear it under his clothes as he did not want anyone to see it. Some felt it would be a good idea to wear them over one layer of clothes so that it would feel more comfortable while still being hidden.

### 3.3. Best Time for Wearing an Accelerometer

There were mixed views as to when the best time to wear accelerometers might be. Some felt that holiday time would be better in order to reduce questioning from peers. However, young people understood that, from a research perspective, wearing the device during school time provided a more accurate account of their activity levels. Pupils from one primary school showed preferences for wearing during term time, whereas those from the other primary school had no preference, highlighting that there are varied views on this subject. One boy aged 9 reported not wanting to wear the accelerometer at school.

### 3.4. Disadvantages of Accelerometers

For those who had had the opportunity to wear an accelerometer prior to the FGD, concerns were raised as to the way it felt and how it might be perceived by their peers. The device was described by some as being “uncomfortable” or “irritating,” especially the elastic belt onto which it was attached. Some also described being embarrassed about having to wear it or felt that it gave other pupils a reason for picking on them, especially if they had weight-related issues.

*“It [accelerometer] gave people an excuse to be more horrible to me.”* (girl, aged 10 with weight-related issues, wore accelerometer 1 week)**

The risk of bullying was therefore heightened among those wearing the accelerometer. A participant with weight-related issues suggested that they would feel being asked to wear an accelerometer as “patronising” and therefore suggested that care should be taken by researchers when introducing the device.

A practical disadvantage of the accelerometer was that children were not able to wear the device for certain activities, such as swimming. Indeed, questionnaire data (not presented in this paper) showed swimming to be among one of the most popular activities amongst the young people in this study. One young person described how he was not able to wear his accelerometer during rugby because of the danger in a contact sport. The device used in this study (GT1M) had a marker button which would allow marking the start of an event, such as a run. The young people reported that they were constantly pressing the button and wondering whether doing so might affect readings. They suggested it would be better to have a device without buttons, to reduce temptation to press. Activity diaries are commonly used alongside accelerometers to record what activities the participant is taking part in. However, when questioned, younger boys (aged 7–11 years) reported that they would not use the diary. Girls were more positive about the thought of completing an activity diary, but they admitted that they would be likely to forget and that it would largely depend on what the activity diary looked like.

### 3.5. How Accelerometers Can Be Made More Appealing/Incentives for Use

Young people made several suggestions as to how accelerometers could be made more appealing or how incentives might be used to improve compliance. All age groups felt that a clip mechanism which fitted onto a child's own belt or clothes would be preferable to an elastic belt. Other suggestions included incorporating the accelerometer as part of a watch, sweatband, or bracelet. The way in which the device looked, in particular its colour, was an important consideration. The option of choosing from a variety of colours was thought to be a good way of making it more appealing. Participants also suggested that being able to personalise the device would encourage its use, for example, using stickers or having the young person's name or school on it. A ‘‘clip-on” cover, similar to those available for mobile phones, was suggested as a way of personalising. Offering tangible rewards, such as money, was also mentioned as a way to encourage young people to wear accelerometers.

* “I would be happy to wear [accelerometer] if paid.”* (boy, aged 15; handled accelerometer at start of focus group)**

Across the age groups a major incentive put forward for wearing the accelerometer was being able to have feedback on their own activity levels, as this would provide worth and encouragement. However, it was also acknowledged that this might encourage “cheating” or asking friends to wear the accelerometer. Young people felt that in order to address this, it would be better to provide accelerometers to the whole class rather than just certain individuals. Doing this may also prevent an individual from feeling singled out.

Younger children felt that it would be good for researchers or teachers to discuss with the whole class the reasons for wearing an accelerometer if a child in that class was going to wear one. This would reduce curiosity from other children. However, it was also acknowledged that this might bring unwanted attention to the child wearing the accelerometer and even encourage bullying or make the child feel different. All young people said they would feel comfortable discussing their accelerometer in front of a class of peers but embarrassed in front of older children in the school. Giving parents an accelerometer to wear alongside their children was also suggested, making accelerometer wearing a whole family activity and hence encouraging children to wear one. Having role models (such as sports people) wearing accelerometers might help further encourage its use. Young people recommended that wearing the accelerometer should be presented by the researcher in a positive way. For example, how it may benefit the child, explaining how it could encourage them and other children to stay healthy, and fit. Introducing the importance of the accelerometer as a device for seeing how fit, healthy and active a person is was suggested. It could be emphasised how those young people wearing the accelerometers are helping research and potentially helping other young people. Young people also felt they would feel more comfortable about wearing an accelerometer if they knew that others were also wearing one.

## 4. Discussion

To our knowledge, there has been little focus on young people's views on wearing accelerometers in physical activity research [15]. Of the research available, it has been suggested that compliance may be increased with the use of rewards, personal activity graphs, and less obtrusive belts and monitors [12]. This small scale user-involvement study adds to initial data in this area, confirming some already highlighted issues, as well as introducing further areas for consideration. This paper provides practical issues for researchers to consider when embarking on accelerometer research among young people. Based on the current study findings, we make a number of suggestions to researchers at the design stage of a study involving young people and accelerometers. These suggestions are focussed around introducing the accelerometer, practical issues, individual factors associated with wearing an accelerometer, and incentives for compliance (Table 2).

Young people's reports of their first impressions of the accelerometer highlight the need for introducing the accelerometers in a clear and informative way. Furthermore, this study begins to reveal that individual factors such as gender, weight status, or current physical activity behaviours may affect an individual's likelihood of wearing the device. It highlights the need to make judgements on an individual participant basis. As suggested in Table 2, a trial wearing of the device (e.g., 24 hours) may highlight issues that can then be addressed during this time and potentially increase the likelihood of compliance during the study period. Practicalities of a “trial period” will of course depend on the study size and duration and number of participants. Previous studies have suggested that accelerometer measures tend to be higher on the first day of recording [22]. Some young people in the current study did report that it took them a little to get used to wearing the device. As such, a period of habituation once a participant has started to wear an accelerometer for data collection is advised.

The current study begins to reveal that individuals with weight-related issues may have certain opinions about the use of an accelerometer that others may not, for example, being at an increased risk of bullying. As current literature on young people's views on accelerometer use is limited, this study highlights an even greater need for understanding the needs and concerns of overweight/obese children in this context.

Previous findings have demonstrated how young people's physical activity patterns can vary between school days and weekends [23] and time of year [24]. The time at which the device is worn is, therefore, likely to have an impact. There were mixed findings as to whether young people preferred wearing accelerometers during school term or school holidays. For research studies which are measuring change in physical activity over time, it is important that longitudinal measurements are carried out either in school term or school holidays, if permitted by the study protocol, in order to minimise intraindividual variation. Giving children the choice of school term or school holidays may help compliance.

Previous research on adolescent perspectives on accelerometer use has been conducted in large-scale population-based school studies [12]. While this is a common setting for accelerometer based research [6], studies involving community-based interventions (e.g., family-based obesity treatment interventions) are likely to involve one-to-one communication with participants, perhaps within their home or a community venue. As such, different issues may be present compared with population-based research. For example, the overweight child is being singled out to wear the accelerometer and is likely to be the only one in their class. This may present specific challenges to the researcher to minimise the risk of bullying. Strategies could include offering other children in the child's class the opportunity to have their physical activity measured and support to the child to help them explain the monitor to their classmates. The allocation of accelerometers to children is more likely to be on a one-to-one level between the researcher and participant and therefore offers the potential for more time to be invested in individual concerns. Providing activity graphs [12] may therefore be easier in this context and was certainly an incentive which participants in the current study supported.

Although not within the control of the researcher, accelerometer manufacturers may want to consider the aesthetics of devices, with the suggestion from children that clip-on covers, such as those used on mobile phones, may be a valuable option for increasing its appeal and ultimately compliance. These are extra costs for the researcher but may improve the quality of the data being collected.

This is a small-scale study within a specific group of young people in the West Midlands, England. As such, the study findings are limited to a small group of individuals which are not necessarily representative of young people within different communities. However, initial findings are revealing and certainly warrant further exploration. These findings are to be used in a current randomised controlled trial of the “Families for Health” programme, an intervention for overweight and obese children and their families in Coventry, Warwickshire, and Wolverhampton.

## 5. Conclusions

In conclusion, this user-involvement study provides initial data on young people's views on accelerometer use in physical activity research. Children report both positive and negative aspects of wearing accelerometers. While positive aspects relate to feeling “special” and having increased attention from friends, negative aspects include the size and comfort of the device as well as an increased risk of bullying. Young people offered advice on how to make wearing accelerometers more appealing, including presenting the device in a positive way, using a clip rather than elastic belt to attach it, personalising the device, and having feedback on activity levels. Judgements over the way in which accelerometers are used should be made at the study development stage and based on the individual population. Further research in this area is suggested, in particular exploring the views of specific populations, such as overweight/obese children or those attending a community health intervention.

## Figures and Tables

**Table 1:** Details of focus group discussion participants/topic.

Group	age (years)	*N* (wore accelerometer)	Time worn accelerometer	Focus group discussion topic
1 (MCRN)	10–18	10 (0) (1 boy, 9 girls)	Handled at start of focus group	Views on accelerometers
2 (MCRN)	8–17	10 (3) (5 boys, 5 girls)	5 hours	Unique group formed to consult on a proposed obesity interventional RCT discussed both the design of a physical activity questionnaire and views on accelerometer use
3 (School)	8–10	3 (3) (1 boy, 2 girls)	One week	Feedback on accelerometer use
4 (School)	7–11	6 (6) (3 boys, 3 girls)	5 hours	Feedback on accelerometer use
5 (School)	7–11	6 (6) (3 boys, 3 girls)	5 hours	Feedback on accelerometer use

**Table 2:** Practical advice for researchers using accelerometers for physical activity research with young people.

Introducing the accelerometer	
(i) For school-based studies, introduce the research to the whole class. This may help reduce curiosity of others and reduce the risk or bullying	
(ii) Providing alternative “placebo” devices (e.g., pedometer) to other pupils may help in involving all pupils and reduce stigma	
(iii) Explain to participants that the accelerometer is not “watching” them, that is, there is no camera or microphone, and only the researcher will see the results. This is particularly important for younger children. Showing participants an example of what these data look like (e.g., a graph) may help them to understand what is being recorded	
(iv) Consider the use of “role models” to promote the wearing of an accelerometer	
(v) Present the accelerometer in a positive way, for example, by wearing it, the participant is helping us to understand how they and other children can live healthier, happier lives	
(vi) Consider a trial wearing of the device (e.g., 24 hours)/period of habituation prior to data collection	

Practical issues	
(i) Use a clip rather than elastic band to attach the accelerometer	
(ii) Where possible, choose a small accelerometer without extra buttons*	
(iii) Certain popular activities, such as swimming or rugby, cannot be recorded due to practicalities of wearing the accelerometer at these times. This highlights the need for an activity diary, not only to record data on the accelerometer use, but also at times when it cannot be used. Diary use compliance is however low, and therefore alternative ways of gaining these data should be explored	

Individual factors associated with wearing an accelerometer	
(i) Judgements over the way in which an accelerometer is used should be made on an individual participant basis	
(ii) Discuss with the child what would make them feel more comfortable wearing the device (e.g., under or over clothes, timing, e.g., term time or holiday time) and administer accordingly where study protocol permits	

Incentives for compliance	
(i) Provide small stickers to personalise the accelerometers. These could be supplied at the same time as the accelerometer for the children is uses and removed once the child returns the device	
(ii) Unlike the pedometer, participants are not able to instantly view their activity levels. Therefore, provide an incentive to children for wearing the accelerometer for the whole study period. This could be in the form of feedback about their activity levels, as well as a certificate for wearing it for the full time	
(iii) Provide the parent with an accelerometer at the same time and ask them to wear it at the same time as their child, in order to make it a family activity and potentially increase compliance	

*The new Actigraph model GT3X is smaller than the one used in this study (GT1M) and is free of buttons.
